# Evaluation of shear bond strength of orthodontic brackets using trans-illumination technique with different curing profiles of LED light-curing unit in posterior teeth

**DOI:** 10.1186/2196-1042-14-49

**Published:** 2013-11-21

**Authors:** Farzin Heravi, Saied Mostafa Moazzami, Negin Ghaffari, Javad Jalayer, Yasaman Bozorgnia

**Affiliations:** Dental Materials Research Center, Mashhad University of Medical Sciences, Mashhad, 91735-984 Iran; Department of Operative Dentistry, Mashhad University of Medical Sciences, Mashhad, 91735-984 Iran; Mashhad University of Medical Sciences, Mashhad, 91735-984 Iran; Department of Orthodontics, North Khorasan University of Medical Sciences, Bojnurd, 9414974877 Iran

**Keywords:** Bond strength, Orthodontic brackets, Light cure adhesive, Pulp chamber

## Abstract

**Background:**

Although using light-cured composites for bonding orthodontic brackets has become increasingly popular, curing light cannot penetrate the metallic bulk of brackets and polymerization of composites is limited to the edges. Limited access and poor direct sight may be a problem in the posterior teeth. Meanwhile, effectiveness of the trans-illumination technique is questionable due to increased bucco-lingual thickness of the posterior teeth. Light-emitting diode (LED) light-curing units cause less temperature rise and lower risk to the pulpal tissue. The purpose of this study was to evaluate the clinical effectiveness of trans-illumination technique in bonding metallic brackets to premolars, using different light intensities and curing times of an LED light-curing unit.

**Methods:**

Sixty premolars were randomly divided into six groups. Bonding of brackets was done with 40- and 80-s light curing from the buccal or lingual aspect with different intensities. Shear bond strengths of brackets were measured using a universal testing machine. Data were analyzed by one-way analysis of variance test and Duncan's *post hoc* test.

**Results:**

The highest shear bond belonged to group 2 (high intensity, 40 s, buccal) and the lowest belonged to group 3 (low intensity, 40 s, lingual). Bond strength means in control groups were significantly higher than those in experimental groups.

**Conclusions:**

In all experimental groups except group 6 (80 s, high intensity, lingual), shear bond strength was below the clinically accepted values. In clinical limitations where light curing from the same side of the bracket is not possible, doubling the curing time and increasing the light intensity during trans-illumination are recommended for achieving acceptable bond strengths.

## Background

Acid etch technique was first introduced by Buonocore in 1955 [1], and since then, it has become increasingly popular among dentists due to its numerous advantages. It has been introduced by Newman [2] in the field of orthodontics and used for direct bonding of brackets for many years.

Using self-polymerizing composites for bonding brackets needs significant time for mixing, so it is a time-consuming procedure when multiple mixes are required. The polymerizing process begins immediately after mixing; therefore, limited time is available for positioning the brackets. Another disadvantage of this method is the possibility of air bubble lockup into the adhesive during mixing that would reduce the bond strength of orthodontic brackets. However, using light-polymerizing composites provides extended working time for prompt bracket positioning and easier residue removal [3]. Moreover, it has been demonstrated that the initial bond strength of brackets would be higher by using light-polymerizing composites rather than self-polymerizing materials [4].

Since curing light cannot penetrate the metallic bulk, polymerization of composites under metallic brackets is limited to the edges of the bracket base. So it may result in incomplete polymerization and diminished bond strength [3]. Some researchers have suggested that curing the composite layer under metallic brackets can be performed from mesial and distal aspects for 20 s each [5]. However, limited access to and poor direct sight of posterior segments may cause some difficulties especially when curing the composite under the bracket from the distal aspect. Therefore, trans-illumination technique has been proposed to cure the composite under metallic brackets by Tavas and Watts in 1979 [6]. In this technique, light is emitted from the opposite side of the tooth and passes through bucco-lingual thickness toward the composite under the metallic bracket. Although little information can be found in orthodontic literature to confirm its usefulness, there is a general agreement on increasing the curing time while using this technique [7]. King et al. [8] tripled the trans-illumination curing time and found proper shear bond strength values regardless of the bucco-lingual thickness of the teeth ranging from 3.4 to 7 mm. Oesterle and Shellhart [7] used human maxillary incisors and found out that the bond strength of 50-s curing from the lingual aspect was so close to the control group and there was no significant difference in adhesive remaining index between control and experimental groups.

There are various types of light-curing units with different spectral profiles and light intensities. While low-intensity light may lead to inadequate depth of cure and insufficient bond strength, high-intensity light might cause excessive heat during irradiation [9–11]. When using trans-illumination technique, the concerns over pulp chamber temperature rise become more important because the light passes through the bucco-lingual thickness of the tooth including the pulp chamber. Many studies have been done to compare pulp chamber temperature rise during polymerization while using different types of light-curing units. Yazici et al. [12] and Haiduc et al. [13] reported that using light-emitting diode (LED) light-curing unit results in significantly lower temperature rise in comparison with conventional halogen units.

As in some cases, it is proposed to have a trans-illumination for bonding of posterior brackets due to limited access from the buccal side to their distal aspect and also for being assured from complete polymerization of bonding materials under the metallic brackets. The purpose of this study was to evaluate the effectiveness of trans-illumination technique in bonding metallic brackets to premolars while using different curing profiles of LED light-curing unit.

## Methods

Based on the results of Oesterle and Shellhart's study [7] and the following formula, the sample size was found to be 10:


where *n* is the sample size, *N* is the population size, *δ*^2^ is the variance, and *B* is the error bound. To estimate *δ*^2^, we used an elementary sample with *α* = 0.05. The power of the study was measured to be 80%. Sixty human premolars that were recently extracted for orthodontic purposes were collected. The criteria for tooth selection were intact buccal enamel, no pretreatment of chemical agents (such as derivatives of peroxide, acid, or alcohol), no cracks from forceps, no caries, and no restorations.

The teeth were sterilized in buffered formalin as Lee et al. suggested in 2007 [14]. One week prior to use, the teeth were placed in isotonic normal saline (0.90% *w*/*v* of NaCl, 300 mosM/L) in order to avoid any possible effect of the remaining solution on the bonding process. The storage media was changed every day to avoid bacterial growth. The teeth were embedded in auto-polymerizing polymethyl methacrylate. A mounting jig was used to align the facial surface of the teeth to be perpendicular to the bottom of the mold and its labial surface parallel to the force during the shear bond strength (SBS) test.

Before bonding, the teeth were randomly divided into six groups each containing ten teeth. Specimen preparation was done exactly as was instructed by the manufacturer. The labial surfaces of the teeth were polished using non-fluoride pumice and then rinsed with water and subsequently dried with moisture-free air. The buccal enamel was etched with a 37% phosphoric acid (UltraEtch, Ultradent Products Inc., South Jordan, UT, USA) for 30 s. Afterwards, the etched surface was rinsed for at least 15 s until the etchant was completely removed and then dried with oil and moisture-free air source. A thin uniform coat of Transbond XT primer (3 M Unitek, Monrovia, CA, USA) was applied to the etched surfaces. A small amount of adhesive paste of Transbond XT was applied to the bracket bases. Stainless steel brackets used in this study were Ultratrim Standard Edgewise (Dentaurum, Ispringen, Germany) in all groups. Immediately after placing the adhesive, the brackets were lightly placed on the tooth surface, adjusted to the final position and then pressed firmly. Excess adhesive material was gently removed from around the bracket base without disturbing it.

The adhesive was cured with High Power (800 mW/cm^2^) and Low Power (650 mW/cm^2^) programs of Bluephase C8 (Ivoclar, Vivadent, Schaan, Liechtenstein) LED-curing unit in all groups:

- Group 1 (control): 40 s light curing with the Low Power program from the buccal aspect (10 s for each mesial, distal, occlusal, and gingival aspect).
- Group 2 (control): 40 s light curing with the High Power program from the buccal aspect (10 s for each mesial, distal, occlusal, and gingival aspect).
- Group 3 (experimental): 40 s light curing with the Low Power program from the lingual aspect (light cure tip was placed as close as possible to the lingual surface perpendicular to occluso-gingival axis of the tooth).
- Group 4 (experimental): 80 s light curing with the Low Power program from the lingual aspect (light cure tip was placed as close as possible to the lingual surface perpendicular to occluso-gingival axis of the tooth).
- Group 5 (experimental): 40 s light curing with the High Power program from the lingual aspect (light cure tip was placed as close as possible to the lingual surface perpendicular to occluso-gingival axis of the tooth).
- Group 6 (experimental): 80 s light curing with the High Power program from the lingual aspect (light cure tip was placed as close as possible to the lingual surface perpendicular to occluso-gingival axis of the tooth).

After bonding, specimens were stored individually in a normal saline solution at 37°C in a dark environment 24 h prior to testing. The shear bond strength of specimens was measured using a Zwick testing machine (Zwick GmbH & Co, Ulm, Germany) at a crosshead speed of 0.5 mm/min. In order to avoid bias, the bonding and debonding procedures were done by two different operators and the teeth were given codes unrelated to their group numbers. The results of the SBS test were recorded in megapascal.

### Statistical analysis

The data was confirmed to be normally distributed using the Kolmogorov-Smirnov test. One-way analysis of variance (ANOVA) and Duncan's *post hoc* tests were used to compare SBS values among groups using SPSS 11.5 software.

## Results

The one-way ANOVA test showed that there was a significant difference among shear bond strength amounts in groups (*F*(5,10) = 719.394, *p* < 0.05). Also, as it is shown in Table 1, Duncan's *post hoc* tests revealed that there was a significant difference between every two groups except for groups 1 and 2 (control group).

**Table 1:** **Duncan's** ***post hoc*** **test**

Group	***N***	Subset for ***α*** = 0.05				
		1	2	3	4	5
Group 1	10					13.6120
Group 2	10					13.9920
Group 3	10	2.9190				
Group 4	10		3.5210			
Group 5	10			4.9590		
Group 6	10				8.4420	
Significance		1.000	1.000	1.000	1.000	0.151

Group 1 (control): 40 s, 650 mW/cm^2^, buccal; group 2 (control): 40 s, 800 mW/cm^2^, buccal; group 3: 40 s, 650 mW/cm^2^, lingual; group 4: 80 s, 650 mW/cm^2^, lingual; group 5: 40 s, 800 mW/cm^2^, lingual; group 6: 80 s, 800 mW/cm^2^, lingual. The test showed that there was a significant difference between every two groups except for groups 1 and 2 (control groups).

Mean values of SBS are shown in Table 2. The highest mean bond strength was seen in group 2 (40-s curing from the buccal aspect with the intensity of 800 mW/cm^2^) and the lowest value was in group 3 (40-s curing from the lingual aspect with the intensity of 650 mW/cm^2^).

**Table 2:** **Shear bond strength values in all six groups**

	***N***	Mean (MPa)	Standard deviation	Standard error	Minimum (MPa)	Maximum (MPa)
Group 1	10	13.6	0.44	0.13	13.0	14.1
Group 2	10	13.9	0.54	0.17	13.4	14.8
Group 3	10	2.9	0.34	0.11	2.4	3.5
Group 4	10	3.5	0.37	0.11	2.9	3.9
Group 5	10	4.9	0.62	0.19	4.1	6.1
Group 6	10	8.4	0.94	0.29	7.2	9.5
Total	60	7.9	4.59	0.59	2.4	14.8

Group 1 (control): 40 s, 650 mW/cm^2^, buccal; group 2 (control): 40 s, 800 mW/cm^2^, buccal; group 3: 40 s, 650 mW/cm^2^, lingual; group 4: 80 s, 650 mW/cm^2^, lingual; group 5: 40 s, 800 mW/cm^2^, lingual; group 6: 80 s, 800 mW/cm^2^, lingual. The highest mean bond strength was seen in group 2 and the lowest value was in group 3.

SBS was not significantly different between two control groups; in both groups, the SBS values were above the clinically accepted values according to Reynolds' study [15], and values were significantly higher than experimental groups.

Among four experimental groups, the only group with sufficient SBS values for orthodontic bonding was group 6 (80-s curing from the lingual aspect with the intensity of 800 mW/cm^2^).

## Discussion

Tavas and Watts [6] first introduced trans-illumination technique in 1979, which suggested curing composite adhesive under metallic brackets from the opposite side of the tooth. Many studies have been done on this technique mostly on the anterior teeth, and the effectiveness of this technique has been proved in the anterior region. King et al. [8] studied trans-illumination technique using bovine teeth and lingual brackets. They tripled the curing time and found proper shear bond strength values when using trans-illumination regardless of the bucco-lingual thickness of the teeth ranging from 3.4 to 7 mm. Oesterle and Shellhart [7] used human maxillary incisors and compared shear bond strength when curing from the buccal (as control groups) or lingual (trans-illumination technique) aspect. They found bond strength values very close to control groups with 50-s curing from the lingual aspect and no statistical difference in adhesive remaining index between control and experimental groups.

Although in posterior segments, limited access and poor direct sight cause some difficulties in curing the adhesive from the same side of the bracket (especially when it is needed to be cured from the distal or gingival aspect), there is little in orthodontic literature about the effectiveness of trans-illumination technique in posterior segments. Regardless of the conclusion of each study, there is a general agreement on using higher light energy (light intensity or curing time) while directing the light through the bucco-lingual thickness of the tooth [7]. However, possible damaging effects of excessive heat generated during polymerization have been warned [9–11]. Previous studies have shown increase in temperature ranging from 1.5°C to more than 4°C in the pulp chamber of extracted teeth [9, 10] which were assumed to be caused by both exothermic reaction and energy absorbed during irradiation [16–20]. The critical temperature for making irreversible damage to the pulpal tissue is 42°C to 42.5°C [21, 22]. As Yazici et al. [12] and Haiduc et al. [13] demonstrated in their studies, using LED units can cause significantly lower temperature rise in the pulp chamber in comparison with halogen units. Consequently, in our study, a polywave LED unit, Bluephase C8 (Ivoclar, Vivadent, Schaan, Liechtenstein), was used in bonding procedures in order to make minimum temperature changes in the pulp chamber.

As it is shown in Table 2, the highest shear bond strength was in group 2 (40-s curing from the buccal aspect with the intensity of 800 mW/cm^2^) and the lowest value was in group 3 (40-s curing from the lingual aspect with the intensity of 650 mW/cm^2^).

Increase in light intensity from 650 to 800 mW/cm^2^ between the control groups did not result in any significant increase in SBS values. Among the four experimental groups, the only group with sufficient SBS values for orthodontic bonding was group 6 (80-s curing from the lingual aspect with the intensity of 800 mW/cm^2^). In group 4, increasing the curing time in comparison with group 3 resulted in a significant increase in bond strength, but according to Reynolds' study [15], the values were not acceptable for orthodontic treatment. Also, in group 5, increasing the intensity (without increasing curing time) in comparison with group 3 resulted in higher bond strengths, but still the values were not acceptable. This leads to the deduction that doubling the light curing time and increasing the light intensity to 800 mW/cm^2^[2] have to be done simultaneously in order to achieve acceptable bond strength values when using trans-illumination technique.

In another similar study on human premolars using metallic and ceramic orthodontic brackets with different curing times of a halogen unit [23], SBS in control groups (cured from the buccal aspect) was significantly higher than that in experimental groups (cured from the lingual aspect), and in one of their experimental groups with 40-s curing from the lingual aspect, the SBS values were lower than the values accepted for clinical orthodontic treatment as they were in our study. On the other hand, in the other experimental groups of their study, SBS values were clinically acceptable. This difference between the results can be the consequence of the difference in light-curing units used in each study.

## Conclusions

According to the results of this study, using trans-illumination technique in bonding metallic brackets to the premolar teeth (in comparison with light curing from the same side of the bracket) resulted in significantly lower bond strengths. In clinical limitations where light curing from the same side of the bracket is not possible, doubling the curing time and increasing the light intensity to 800 mW/cm^2^ during trans-illumination with the LED light-curing unit can be done for attaining acceptable bond strength with minimum risk of overheat to the pulpal tissue.
